# Impact of comorbid polycystic ovarian syndrome and gestational diabetes mellitus on pregnancy outcomes: a retrospective cohort study

**DOI:** 10.1186/s12884-020-03175-5

**Published:** 2020-08-24

**Authors:** Varun Manoharan, Vincent W. Wong

**Affiliations:** 1 Diabetes and Endocrine Service, Liverpool Hospital, Sydney, NSW Australia; 2 South Western Sydney Clinical School, University of New South Wales, Sydney, NSW Australia

**Keywords:** Gestational diabetes mellitus, Polycystic ovarian syndrome, Pregnancy, Outcomes, Complications

## Abstract

**Background:**

Gestational diabetes mellitus (GDM) and polycystic ovarian syndrome (PCOS) have been associated with adverse maternal and neonatal outcomes, but the evidence on the impact of coexistent PCOS and GDM is rather limited and inconclusive. We investigated the impact of comorbid PCOS on pregnancy outcomes among women with GDM.

**Methods:**

This retrospective cohort study included women diagnosed with GDM on 75 g oral glucose tolerance test on routine antenatal screening tests at Liverpool Hospital between February 2015 and January 2019. Women were then grouped into those with and without PCOS based on the Rotterdam criteria. The demographic details, clinical data and pregnancy outcomes were compared between the two groups.

**Results:**

Among the 1545 women with GDM included in the study, there were 326 women with PCOS. Women with GDM and PCOS (GDM^+^PCOS^+^) were younger (29.5 years vs 31.5 years, *p* < 0.001), more likely to be primigravidae (31.9% vs 20%, *p* < 0.001), as well as of Caucasian descent (37.4% vs 21.7%, *p* < 0.001). PCOS was an independent risk factor for the development of preeclampsia on regression analysis (OR 2.06, *p* = 0.021). Women with PCOS and GDM had a higher body mass index (31.5 kg/m^2^ vs 27.7 kg/m^2^, *p* < 0.001), significant gestational weight gain (12.6 kg vs 11.5 kg, *p* = 0.016), and more frequent use of pharmacotherapies to manage their GDM (57.7% vs 45.2%, *p* < 0.001). There was no statistically significant difference in the rates of adverse neonatal outcomes in both the groups.

**Conclusion:**

Among women with GDM, PCOS was an independent risk factor for the development of preeclampsia and significant gestational weight gain, warranting vigilant monitoring of blood pressure, blood glucose levels and body weight, and implementing timely interventions to improve obstetric and neonatal outcomes.

## Background

Polycystic ovarian syndrome (PCOS) is the most common endocrine disorder that affects women of reproductive age with a prevalence of 12–21% [1], and it has a profound impact on the psychological, reproductive and metabolic health of women [2]. The Rotterdam criteria, the most widely used criteria for the diagnosis of PCOS, encompasses any two of the following three criteria: ovulatory dysfunction, sonographic evidence of polycystic ovarian morphology, and biochemical evidence or clinical features associated with hyperrandrogenism [3]. Women with PCOS have a higher risk of developing type II diabetes (T2DM) [4–6]. Some [7, 8], but not all [9–11], studies suggest that PCOS increases the risk of developing GDM. Insulin resistance is a common metabolic aberration in GDM [12] and PCOS [13, 14]. Both GDM [15, 16] and PCOS [17, 18] have been associated with adverse maternal and neonatal outcomes. The evidence on the maternal, pregnancy and neonatal outcomes among women with coexistent PCOS and GDM (GDM^+^PCOS^+^) is rather limited and inconclusive [19–22].

The study aims to retrospectively evaluate the impact of comorbid PCOS on pregnancy outcomes among with women with GDM. The study is explorative and aims to contribute to the existing literature on vigilant monitoring of blood glucose levels and implementing timely interventions among pregnant women with PCOS and GDM in order to improve obstetric and neonatal outcomes.

## Methods

This retrospective cohort study was performed on women diagnosed with GDM on routine antenatal screening with the 75 g oral glucose tolerance test (OGTT) at Liverpool Hospital between February 2015 and January 2019. We defined GDM as fasting plasma glucose level greater than 5.1 mmol/L, one hour plasma glucose level greater than 10.0 mmol/L and two hour plasma glucose level greater than 8.5 mmol/L as per the consensus guidelines released by the Australasian Diabetes in Pregnancy Society [23]. All women completed a standardised, self-report questionnaire during their first antenatal visit, which included demographic information, medical history, obstetric history and psychosocial history (Supplement 1). The medical history included a question about previous diagnoses of PCOS. Women who reported to have PCOS were confirmed about their diagnosis based on the Rotterdam criteria. Demographic, clinical and follow-up data were gathered through the South Western Sydney Local Health District (SWSLHD) electronic medical record system. Clinical variables of interest included obstetric history, anthropometric data, glycaemic parameters in the index OGTT, antenatal complications such as the incidence of preeclampsia, and neonatal complications including macrosomia, neonatal hypoglycaemia, and admission of the neonate to the neonatal intensive care unit.

The GDM database project was approved by the SWSLHD Human Research Ethics Committee. Patient consent was waived in accordance to the ethics proposal approved by the SWSLHD Human Research Ethics Committee on the basis that the project was deemed to be of low and negligible risk, and that the retrospective consent of 1545 women enrolled in this study would have placed undue burden on those involved in this research.

Statistical analyses were performed using IBM SPSS Statistics 21. Absolute numbers and percentages were used to describe the patient population. Normal distribution of continuous variables was tested using Kolmogorov-Smirnov and Shapiro-Wilk tests. Quantitative variables that were normally distributed are expressed as mean and standard deviation. Where the quantitative variables were not normally distributed, the values are presented as median and range. Independent two sample *t*-test was used to analyse continuous variables, and chi-square test was used to examine associations between categorical variables. Logistic regression testing was used to adjust for confounding variables. A *p* < 0.05 was considered statistically significant.

## Results

Among the 1545 women with GDM included in the study, there were 326 (21.1%) women with PCOS. Women with GDM and PCOS (GDM^+^PCOS^+^) were younger (29.5 years vs 31.5 years, *p* < 0.001) and more likely to be primigravidae (31.9% vs 20%, *p* < 0.001), current smokers (11.1% vs 7.2%, p < 0.001) as well as of Caucasian descent (37.6% vs 21.8%, *p* < 0.001). Anthropometrically, GDM^+^PCOS^+^ women recorded a higher body mass index (31.5 kg/m^2^ vs 27.7 kg/m^2^, *p* < 0.001) and a statistically significant gestational weight gain (12.6 kg vs 11.5 kg, *p* = 0.016).

In our study, GDM^+^PCOS^+^ women had a lower incidence of previous GDM (18.9% vs 36.3%, *p* < 0.001) with nulliparous removed from this analysis. There was a significantly higher usage of metformin (20.5% vs 12.4%, *p* < 0.005) and insulin (57.1% vs 44.1%, *p* < 0.001) in these women with a small proportion of women requiring a combination of metformin and insulin (5.4% vs 4.3%). Although GDM^+^PCOS^+^ women had a higher mean fasting blood glucose level (BGL) on OGTT (5.4 mmol/L vs 5.1 mmol/L, *p* = 0.006), there were no significant differences in the other glycaemic parameters, including the mean BGL at 1 h and 2 h, and the HbA1c at diagnosis of GDM (Table 1).

**Table 1:** Patient characteristics, anthropometric profile and glycaemic profile

	GDM^**+**^PCOS^**+**^ ***N*** = 326	GDM^**+**^PCOS^**0**^ ***N*** = 1215	***p***-value
Mean age (years)	29.5 (±4.4)	31.5 (±5.3)	<0.001
Ethnicity			<0.001
Caucasian	124 (37.8)	266 (21.8)	
Non Caucasian	204 (62.2)	951 (78.2)	
Africans	6 (1.8)	40 (3.2)	
Southeast Asians	30 (9.2)	257 (21.2)	
South Asians	42 (12.8)	226 (18.6)	
Pacific Islanders	16 (4.8)	88 (7.3)	
Middle-East	96 (29.4)	312 (25.7)	
South Americans	4 (1.2)	15 (1.2)	
Indigenous	6 (1.8)	10 (0.8)	
Other	4 (1.2)	3 (0.2)	
Gravidity			<0.001
Primigravidity	104 (31.9)	244 (20)	
Multigravidity	222 (68.1)	973 (80)	
<5 pregnancies	180 (55.2)	740 (60.1)	
≥5 pregnancies	42 (12.9)	233 (19.1)	
Smoking status			0.020
Smokers	36 (11.1)	87 (7.2)	
Non-smokers	288 (88.9)	1129 (92.8)	
Family History of diabetes			0.950
Yes	150 (46.6)	562 (46.8)	
No	172 (53.4)	639 (53.2)	
Past history of GDM^a^			<0.001
Yes	32 (18.9)	305 (36.3)	
No	138 (81.1)	536 (63.7)	
Mean Body Mass Index (kg/m^2^)	31.5 (±6.7)	27.7 (±9.4)	<0.001
Body Habitus			<0.001
Non-obese	200 (62.5)	836 (68.5)	
Obesity (BMI>30)	120 (37.5)	384 (31.5)	
Mean gestational weight gain (kg)	12.6 (±7.7)	11.5 (±7.4)	0.016
Previous insulin use			0.357
Yes	30 (9.2)	144 (11.8)	
No	296 (90.8)	1072 (88.2)	
Use of Metformin during current pregnancy			0.005
Yes	64 (20.5)	151 (12.4)	
No	248 (79.5)	1064 (87.6)	
Insulin used during current pregnancy			<0.001
Yes	178 (57.1)	536 (44.1)	
No	134 (42.9)	679 (55.9)	
Management during current pregnancy			<0.001
Nil medications	132 (42.3)	666 (54.8)	
Metformin only	47 (15.1)	98 (8.1)	
Insulin only	116 (37.2)	399 (32.8)	
Combination	17 (5.4)	52 (4.3)	
Mean dose of prandial insulin at the end of pregnancy (units)	37.7 (±59.5)	35.4 (±39.2)	0.425
Mean dose of basal insulin at the end of pregnancy (units)	18.5 (±23.9)	17.0 (±17.3)	0.529
Mean fasting BGL^b^ on OGTT (mmol/L)	5.4 (±3.6)	5.1 (±0.7)	0.006
Mean 1 hour BGL^b^ on OGTT (mmol/L)	9.7 (±1.7)	9.9 (±1.8)	0.225
Mean 2 hour BGL^b^ on OGTT (mmol/L)	7.7 (±1.7)	7.8 (±2.2)	0.469
Mean HbA1c at diagnosis	5.2 (±0.4)	5.2 (±0.4)	0.539

^a^Nulliparous women were not included in this analysis; ^b^Blood glucose level

In terms of pregnancy outcomes, there was a significantly higher incidence of preeclampsia among GDM^+^PCOS^+^ women (Table 2) compared to those without PCOS. The association between plasma glucose level one hour after 75 g OGTT and the likelihood of preeclampsia was almost significant among GDM^+^PCOS^+^ women (10.4 mmol/L vs 9.4 mmol/L, *p* = 0.051), and not among GDM^+^PCOS^0^ women (9.9 mmol/L vs 9.8 mmol/L, *p* = 0.910). On subgroup analysis, there were no significant associations between the other glycaemic indices and the likelihood of developing preeclampsia in our study population. The proportion of neonates born to GDM^+^PCOS^+^ women with an APGAR score less than 7 at one minute was significantly higher (13.5% vs 8.9%, *p* = 0.021), whilst the incidence of all the other adverse neonatal outcomes, such as APGAR score less than 7 at five minutes, congenital abnormalities, neonatal hypoglycaemia, prematurity, macrosomia, admission to neonatal intensive care unit and neonatal death did not retain statistical significance (Table 2).

**Table 2:** Pregnancy and Neonatal outcomes

	GDM^**+**^PCOS^**+**^	GDM^**+**^PCOS^**0**^	***p***-value
Preeclampsia			0.010
Yes	22 (6.7)	35 (2.9)	
No	304 (93.3)	1180 (97.1)	
Hypertension in pregnancy			0.767
Yes	22 (6.7)	74 (6.1)	
No	304 (93.3)	1144 (93.9)	
Mode of delivery			0.732
Vaginal delivery	218 (66.9)	807 (67.8)	
Caesarean section	108 (33.1)	382 (32.2)	
Vaginal delivery			0.025
Spontaneous onset of labour	102 (46.8)	446 (55.2)	
Induction of labour	116 (53.2)	361 (44.8)	
Caesarean Section			0.004
Emergency C-section	50 (46.3)	120 (31.4)	
Elective C-section	58 (53.7)	262 (68.6)	
Mean week of Delivery	38.47 (30-41)	38.24 (10-42)	0.739
Prematurity^a^			0.753
Yes	32 (9.8)	90 (7.5)	
No	294 (90.2)	1116 (92.5)	
APGAR<7 at 1 mins (%)	13.5	8.9	0.021
APGAR<7 at 5 mins (%)	1.8	2.7	0.547
Mean neonatal birth weight	3309.5 (1342-4545)	3308.5 (1000-5420)	0.982
Macrosomia^b^			0.193
Yes	36 (11)	103 (8.6)	
No	290 (89)	1092 (91.4)	
Admission to NICU			0.556
Yes	42 (12.9)	174 (14.6)	
No	284 (87.1)	1017 (85.4)	
Neonatal hypoglycaemia^c^			0.406
Yes	40 (12.3)	121 (10.2)	
No	286 (87.7)	1071 (89.8)	
Admission to NICU with hypoglycaemia			0.102
Yes	28 (8.6)	72 (6)	
No	298 (91.4)	1120 (94)	
Neonatal death			0.248
Yes	2 (0.1)	17 (0.1)	
No	324 (99.1)	1186 (99.9)	
Congenital abnormality			0.314
Yes	8 (2.5)	33 (2.8)	
Ankyloglossia	4 (1.3)	1 (0.2)	
Cardiovascular	0 (0)	5 (0.4)	
Gastrointestinal	0 (0)	1 (0.2)	
Limb abnormalities	2 (0.6)	14 (1.1)	
Neurological	0 (0)	5 (0.4)	
Genitourinary	2 (0.6)	4 (0.3)	
Other	0 (0)	3 (0.2)	
No	318 (97.5)	1162 (97.2)	

^a^Prematurity is defined as delivery before 37 weeks of gestation; ^b^Macrosomia is defined as birthweight above 4,000g; ^c^Neonatal hypoglycaemia is defined as blood glucose level below 2.5mmol/L within the first 24 hours of birth

## Discussion

A significantly higher proportion primigravidae with GDM had comorbid PCOS (Table 1) and PCOS has been associated with dysglycaemic states such as GDM and T2DM, but the impact of co-existent GDM in women with PCOS on maternal and neonatal outcomes is rather uncertain [19–21]. Our main finding is that GDM^+^PCOS^+^ women had a higher incidence of preeclampsia (Table 2). Interestingly, the rate of pre-existing hypertension was not different between those with or without PCOS, but all the women with pre-existing hypertension in the GDM^+^PCOS^+^ group developed preeclampsia. Women with GDM who developed preeclampsia recorded a significantly higher BMI (32.5 kg/m^2^ vs 28.3 kg/m^2^, *p* = 0.001). Obesity has been associated with a higher risk of developing preeclampsia [22, 24–26], and BMI could confound the association between PCOS and preeclampsia. However, PCOS remained an independent risk factor for the development of preeclampsia on regression analysis (OR 2.06, *p* = 0.021), when adjusted for age, BMI, parity, family history of diabetes mellitus, ethnicity and smoking status. This finding is in keeping with a large meta-analysis conducted by Boomsa et al. [27], in which GDM^+^PCOS^+^ women had more than a five-fold risk of developing preeclampsia; while Foroozanfard et al. [19] found that these women had a 2.8 fold risk of developing preeclampsia. However, Mikola and colleagues [7] demonstrated that PCOS increased the risk of developing GDM and not preeclampsia.

A prospective, randomised, multicenter study showed a statistically significant association between first trimester HbA1c and the incidence of preeclampsia [28]. In our study, women who developed preeclampsia had a significantly higher HbA1c on diagnosis of GDM (5.4% vs 5.2%, *p* = 0.037). Surprisingly, this correlation only retained statistical significance on subgroup analysis among GDM^+^PCOS^0^ women and not in GDM^+^PCOS^+^ women (Table 3). Similarly, the literature also reports a significant correlation between fasting blood glucose level and the likelihood of developing preeclampsia [15], but interestingly, this was also only statistically significant among GDM^+^PCOS^0^ women in our study. These findings not only reflect the complex metabolic aberrations in PCOS, but they also highlight the scarcity of research on pregnancy outcomes in women with GDM with comorbid PCOS.

**Table 3:** Glycaemic parameters in women who developed preeclampsia

	GDM^**+**^PCOS^**+**^			GDM^**+**^PCOS^**0**^		
	PET^a^	No PET^a^	*p*	PET^a^	No PET^a^	*p*
HbA1c at diagnosis (%)	5.2 (±0.5)	5.2 (±0.4)	0.639	5.5 (±0.6)	5.2 (±0.4)	0.045
Mean fasting BGL^b^ on OGTT (mmol/L)	5.2 (±0.4)	5.4 (±3.7)	0.740	5.5(±0.9)	5.1(±0.7)	0.027
Mean 1 hour BGL^b^ on OGTT (mmol/L)	10.4 (±1.6)	9.4 (±1.7)	0.051	9.9 (±2.1)	9.8 (±1.7)	0.910
Mean 2 hour BGL^b^ on OGTT (mmol/L)	7.7 (±1.5)	7.7 (±1.7)	0.991	7.6 (±1.9)	7.8 (±2.1)	0.566

^a^Preeclampsia; ^b^Blood glucose level

Interestingly, among women with GDM and PCOS, we found a statistically significant association between plasma glucose level one hour after 75 g OGTT and the likelihood of preeclampsia was almost significant only among GDM^+^PCOS^+^ women (10.4 mmol/L vs 9.4 mmol/L, *p* = 0.051), and this is in keeping with Joffe et al. [29] who found a significant association between plasma glucose one hour after a 50 g glucose challenge and the likelihood of preeclampsia. On subgroup analysis, there were no significant associations between the other glycaemic indices and the likelihood of developing preeclampsia in our study population (Table 3). These findings highlight the importance of monitoring women with GDM and PCOS for preeclampsia to optimise pregnancy outcomes.

We also found significant differences in the anthropometric parameters between women with and without PCOS. Foroozanfard et al. [19] found no statistically significant difference in the BMI of GDM^+^PCOS^+^ and GDM^+^PCOS^0^ women included in their study, but our analysis showed that obese women with GDM had a higher chance of having comorbid PCOS (OR 2.973, *p* < 0.001) when adjusted for age, parity, ethnicity and past history of GDM, all of which were statistically significant co-variables. Women with GDM and PCOS not only had a higher pre-pregnancy BMI, they also put on more weight during pregnancy (Table 2), and these findings are congruent with the literature [30, 31]. Catalano and Shankar [31] reported long-term implications of gestational weight gain and postpartum weight retention, including an increased risk of venous thromboembolism, depression, difficulty with breast feeding and future cardiovascular risk. Due to the higher BMI at baseline and greater gestational weight gain during pregnancy, there is a greater need to assist these women in terms of weight management in the postpartum period.

We found that GDM^+^PCOS^+^ women had a significantly higher fasting blood glucose level on OGTT, which is in keeping with that of Radon et al. [32] and a meta-analysis conducted by Yu et al. [33] These studies had also shown that maternal obesity was related to fasting glucose levels on OGTT, and we had similar findings on one way ANOVA testing (F (289,1226) = 1.384, *p* < 0.001). Interestingly, on subgroup analysis, we found a statistically significant correlation between BMI of GDM^+^PCOS^+^ women and fasting blood glucose level (F (91,231) = 1.285, *p* = 0.049), one hour plasma glucose level (F(84,205) = 5.048, *p* < 0.001) and two hour plasma glucose level (F(88,223) = 4.120, *p* < 0.001). Among GDM women without PCOS, maternal BMI was only associated with a higher fasting blood glucose level (F(276,915) = 2.296, p < 0.001). These findings highlight the additive impact of PCOS on glucose metabolism among women with GDM, emphasising the importance of monitoring the glycaemic parameters and anthropometric parameters during pregnancy.

There was a significant difference in the management of GDM in women with PCOS, with a higher proportion of them requiring pharmacological intervention (Table 2). A small proportion of GDM^+^PCOS^+^ women in our study required a combination of metformin and insulin (5.4% vs 4.3%), but this is significantly lower compared to 10–44% requirement for supplemental insulin reported by Balani et al. [34] and Rowan et al. [35] On subgroup analysis, GDM^+^PCOS^+^ women who were not on metformin had a significantly higher rate of requiring insulin (OR 37.3, *p* < 0.001) and had higher total insulin requirements at the end of pregnancy (66.9 units vs 26.5 units, *p* = 0.044). Interestingly, on subgroup analysis, the use of metformin among GDM^+^PCOS^+^ women was only associated with lower total insulin requirements (11.3 units vs 75.1 units, *p* = 0.043) among women who were not obese. Our findings are reflective of the complex overlap of metabolic aberrations such as insulin resistance and weight-dependent, altered insulin metabolism associated with obesity and PCOS, which compound the risk of developing GDM. Furthermore, our results highlight the therapeutic role of metformin in the management of insulin resistance caused by PCOS and GDM, but we lack data on whether metformin was commenced preconception. Women who were on metformin pre-conception usually stopped the medication at the end of first trimester, and we are unclear whether the use of metformin in early pregnancy had an impact on the women’s metabolic profile and pregnancy outcomes in the latter part of pregnancy.

The association between PCOS and Caesarean section has been established in the literature [11, 27], but the evidence regarding its incidence in women with PCOS and GDM is rather limited. Foroozanfard et al. [19] reported no significant difference in the incidence of Caesarean section between GDM^+^PCOS^+^ and GDM^+^PCOS^0^ women. However, in our study, even though there was no significant difference in the mode of delivery between the two groups, we found that a higher proportion of women with coexistent GDM and PCOS who had vaginal deliveries required induction of labour and a significantly larger proportion of GDM^+^PCOS^+^ underwent Caesarean section required it as an emergency procedure (Table 2). These findings highlight the importance of vigilantly monitoring these women, especially in the third trimester to plan their delivery and their postpartum care.

The literature also reports a higher incidence of adverse neonatal outcomes among GDM^+^PCOS^+^ women [7, 8]. Neonates born to women with GDM and PCOS had a higher incidence of hypoglycaemia (12.3% vs 10.2%, *p* = 0.406) and admission to NICU with hypoglycaemia (8.6% vs 6%, *p* = 0.102). Similarly, there was a higher incidence of macrosomia among neonates born to GDM^+^PCOS^+^ women (11% vs 8.6%, *p* = 0.193). However, these associations were not statistically significant in our study because our study was not powered for these outcomes.

Interestingly, there was a higher rate of smoking amongst women with PCOS, despite the known association between PCOS and cardiovascular risk factors [31]. This may need to be addressed especially by the primary care physician postpartum to minimise the adverse long-term impact on their cardiovascular health.

Strengths of our study include the recruitment of a large, multicultural and unselected community cohort retrospectively with data collection in real time during follow-ups in the antenatal clinic, which enhances the external validity of the study. Furthermore, we adjusted for statistically significant confounders noted on univariate analysis and confounders reported in the literature to optimise the validity of the data.

We note several limitations in our study. The diagnosis of PCOS was based on a screening question at the time of antenatal booking, and those who self-reported to have PCOS were confirmed about the diagnosis based on the Rotterdam criteria. There is inherent potential for misidentification as there would be women with this condition who were not aware of the diagnosis of PCOS. Furthermore, our study only involved women with GDM; hence we were unable to comment on the impact of PCOS on women who did not develop GDM. The lack of follow-up data relating to post-partum OGTT deterred us from analysing the impact of coexistent GDM and PCOS on the maternal risk of developing T2DM. Owing to the lack of data on the management of PCOS prior to the index pregnancy, especially the duration of metformin therapy, we were unable to evaluate the therapeutic implications of metformin use in PCOS in relation to maternal and foetal outcomes. We also lack data on the use of aspirin in our cohort, which deterred us from analysing the impact of aspirin use on the incidence of pre-eclampsia. Our study was not powered to detect a significant difference among adverse neonatal outcomes associated with GDM, which have been widely reported in the literature.

## Conclusion

In conclusion, in our study that only addresses women with GDM, we found that there were significant differences in demographic, anthropometric and glycaemic parameters in the presence of comorbid PCOS. The coexistence of PCOS among women with GDM was associated with a higher pre-pregnancy BMI and significant gestational weight gain, and comorbid PCOS was an independent risk factor on regression analysis for the development of preeclampsia among women with GDM, all of which have been shown to have long term implications on the health of the mother and the infant. Our findings highlight the importance of regular antenatal follow-up of women with GDM and PCOS, vigilant monitoring of blood pressure and glycaemic control, and better control of gestational weight gain in order to optimise obstetric and neonatal health outcomes.

## Supplementary information

**Additional file 1.** Supplementary file 1. Figure 1 A snapshot of the online antenatal booking sheet. A standardised, self-report questionnaire completed by all women during their first antenatal visit, which includes demographic information, medical history, obstetric history and psychosocial history. The medical history included a question about previous diagnoses of PCOS.

## Data Availability

The datasets generated and/or analysed during the current study are available from the corresponding author on reasonable request.

## Abbreviations

BMI: Body Mass Index
GDM: Gestational diabetes mellitus
PCOS: Polycystic ovarian syndrome
GDM^+^PCOS^+^: Women with gestational diabetes mellitus and polycystic ovarian syndrome
GDM^+^PCOS^0^: Women with gestational diabetes mellitus and no polycystic ovarian syndrome
OGTT: oral glucose tolerance test
SWSLHD: South Western Sydney Local Health District
T2DM: Type II diabetes mellitus
