# Supplementary material for: Impact of comorbid polycystic ovarian syndrome and gestational diabetes mellitus on pregnancy outcomes: a retrospective cohort study
Source: BMC Pregnancy Childbirth. 2020 Aug 24;20:484. doi: 10.1186/s12884-020-03175-5 (PMC7444192; doi:10.1186/s12884-020-03175-5)
Supplement: Supplementary file 1 — Additional file 1. Supplementary file 1. Figure 1 A snapshot of the online antenatal booking sheet. A standardised, self-report questionnaire completed by all women during their first antenatal visit, which includes demographic information, medical history, obstetric history and psychosocial history. The medical history included a question about previous diagnoses of PCOS. [file 12884_2020_3175_MOESM1_ESM.docx]

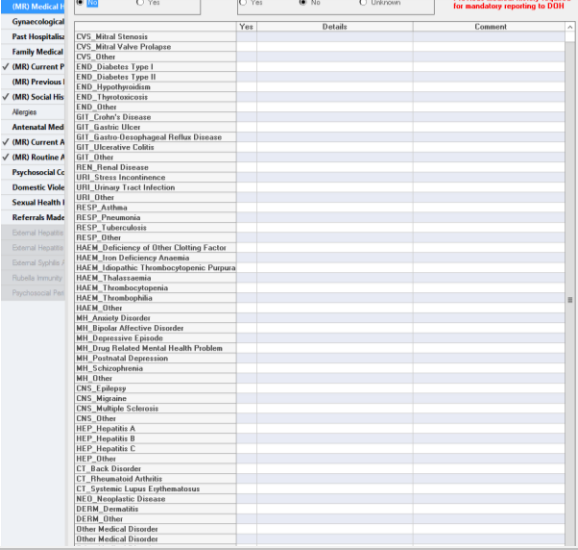


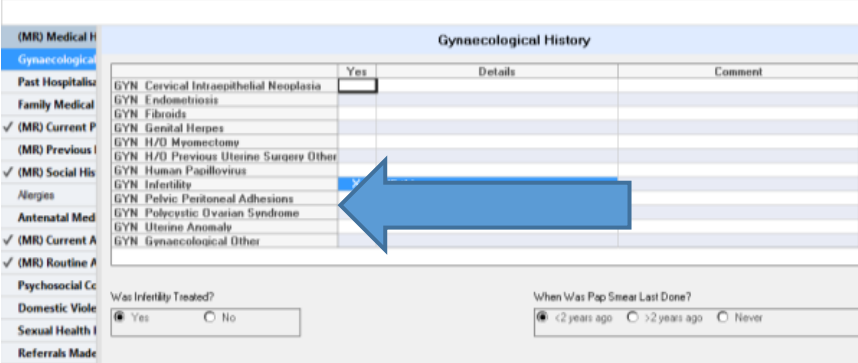


Figure 1. A snapshot of the online antenatal booking sheet.

A standardised, self-report questionnaire completed by all women during their first antenatal visit, which includes demographic information, medical history, obstetric history and psychosocial history. The medical history included a question about previous diagnoses of PCOS. A questionnaire completed by all pregnant women on antenatal booking.
